# Near-Complete Genome Sequence of an Avian Orthoavulavirus Type 13 Strain Isolated in South Korea in 2020

**DOI:** 10.1128/mra.00253-22

**Published:** 2022-06-30

**Authors:** Andrew Y. Cho, Sungsu Youk, Sun-Hak Lee, Tae-Hyeon Kim, Sol Jeong, Yu-jin Kim, Chang-Seon Song

**Affiliations:** a Department of Veterinary Medicine, Konkuk University, Seoul, Republic of Korea; b Southeast Poultry Research Laboratory, U.S. National Poultry Research Center, Agricultural Research Service, U.S. Department of Agriculture, Athens, Georgia, USA; c Wildlife Disease Research Team, National Institute of Wildlife Disease Control and Prevention, Gwangju, Republic of Korea; KU Leuven

## Abstract

We report the near-complete genome sequence of an avian orthoavulavirus 13 (AOAV-13) strain isolated from a wild goose fecal sample collected in South Korea in early 2020. The AOAV-13 sequence had a unique 3′ trailer region, including an 84-nucleotide (nt) deletion and a 24-nt insertion, compared to the most closely related Chinese genome sequence from 2015.

## ANNOUNCEMENT

Avian paramyxoviruses (APMVs) have been reported all around the world, infecting poultry as well as diverse species of wild birds. APMVs belong to the recently assigned subfamily *Avulavirinae* of the family *Paramyxoviridae*. The subfamily *Avulavirinae* is composed of three genera, namely, *Metaavulavirus* (avian metaavulavirus [AMAV]), *Orthoavulavirus* (avian orthoavulavirus [AOAV]), and *Paraavulavirus* (avian paraavulavirus [APAV]) (1). *Avulavirinae* member viruses possess nonsegmented, single-stranded, negative-sense RNA genomes ranging from 14.9 to 17.4 kbp, encoding at least six proteins (NP, P, M, F, HN, and L) (2). In this study, we report the near-complete genome sequence of an AOAV-13 strain that was isolated in South Korea in 2020.

A hemagglutinating agent was isolated from allantoic fluid harvested from 9- to 11-day-old embryonated chicken eggs inoculated with a fecal sample (36°59′14.4″N, 126°39′45.9″E) from a wild migratory bird that had been collected during avian influenza virus surveillance from October 2019 to April 2020. The host species of the isolated virus was determined from wild bird feces by the mitochondrial DNA barcoding PCR technique (3). Viral RNA was isolated using the TRIzol LS reagent. Extracted RNA was amplified using the sequence-independent single-primer amplification (SISPA) method, which utilizes random priming to amplify unknown RNA virus genomes, as described previously (4). Library preparation and sequencing were performed at Biocore Co., Ltd. (Seoul, Republic of Korea), using an Ion Torrent S5TM XL system. The resulting raw reads (1,536,656 reads) were trimmed and mapped to the whole-genome sequence of an AOAV-13 strain from the GenBank database (GenBank accession no. MN150295.1) with Geneious Prime v2021.2.2 using default parameters. A total of 544,667 reads were mapped to produce a near-complete genome of AOAV-13. Putative insertions and deletions identified in the initial assembly were verified with Sanger sequencing with primers designed to amplify the nucleotide position 14508 to 16032 region. The genome positions were calculated based on the AOAV-13/wild goose/China/Hubei/V93-1/2015 (China/V93-1) genome (GenBank accession no. MN150295).

Barcoding PCR of the host mitochondrial DNA identified the host as a greater white-fronted goose (Anser albifrons) by using the primers AvesF (GCATGAGCAGGAATAGTTGG) and AvesR (AAGATGTAGACTTCTGGGTG), as described previously (3). The isolate was named Korea/AOAV-13/Greater white-fronted goose/South Korea/E20-158-3/2020 (here referred to as Korea/E20-158-3). The genome of Korea/E20-158-3 had a GC content of 42.6% (5). A BLAST search of the whole-genome sequence of Korea/E20-158-3 returned the highest identity (99.15%) to China/V93-1. The furin cleavage site of the F gene in Korea/E20-158-3 had the same motif (^112^V-R-E-N-R-L^117^) as found in other AOAV-13 strains. The monobasic amino acid at the C terminus of the cleavage site and leucine at residue 117 suggest low pathogenicity in domestic poultry (2, 6–8). The Korea/E20-158-3 genome sequence had the same length of the 3′ leader region (*n* = 55) as observed in most genomes of the subfamily *Paramyxovirinae* (9). The genome of Korea/E20-158-3 also possessed paramyxovirus-characteristic complementary 5′ leader and 3′ trailer termini of 14 nucleotides (nt) (one mismatch at nucleotide position 9) (9). For the 3′ trailer region, additional Sanger sequencing confirmed that Korea/E20-158-3 exhibited an 84-nt deletion at nucleotide positions 15304 to 15387 and a 24-nt insertion at nucleotide positions 15811 to 15834, compared to the most closely related sequence, China/V93-1. Genome sequencing of Korea/E20-158-3 revealed genomic diversity among AOAV-13 strains.

### Data availability.

The near-complete genome sequence of AOAV-13/Greater white-fronted goose/South Korea/E20-158-3/2020 has been deposited in the GenBank and Sequence Read Archive (SRA) databases under the accession no. OK513542.1 and SRR19361424, respectively.
